# Eyes versus hands: How perceived stimuli influence motor actions

**DOI:** 10.1371/journal.pone.0180780

**Published:** 2017-07-26

**Authors:** Alexander Geiger, Eva Niessen, Gary Bente, Kai Vogeley

**Affiliations:** 1 Institute of Neuroscience and Medicine–Cognitive Neuroscience (INM-3), Research Centre Juelich, Juelich, Germany; 2 Brain Imaging Lab, Department of Psychiatry, University Hospital Cologne, Cologne, Germany; 3 Department of Psychology, Faculty of Human Sciences, University of Cologne, Cologne, Germany; 4 Department of Communication, Michigan State University, Michigan, United States of America; University of Muenster, GERMANY

## Abstract

Many studies showed that biological (e.g., gaze-shifts or hand movements) and non-biological stimuli (e.g., arrows or moving points) redirect attention. Biological stimuli seem to be more suitable than non-biological to perform this task. However, the question remains if biological stimuli do have different influences on redirecting attention and if this property is dependent on how we react to those stimuli. In two separate experiments, participants interact either with a biological or a non-biological stimulus (experiment 1), or with two biological stimuli (gaze-shifts, hand movements)(experiment 2) to which they responded with two different actions (saccade, button press), either in a congruent or incongruent manner. Results from experiment 1 suggest that interacting with the biological stimulus lead to faster responses, compared to the non-biological stimulus, independent of the response type. Results from experiment 2 show longer reaction times when the depicted stimulus was not matching the response type (e.g., reacting with hand movements to a moving object or gaze-shift) compared to a matching condition, while especially the gaze-following condition (reacting with a gaze shift to a perceived gaze shift) led to the fastest responses. These results suggest that redirecting attention is not only dependent on the perceived stimulus but also on the way how those stimuli are responded to.

## Introduction

In everyday life, we are constantly interacting with others, either verbally or nonverbally. For nonverbal communication, we often use our whole body to express what we are thinking or what catches our attention. But sometimes, we only use a subtle movement of a specific body part to send a signal. The question remains, is it crucial how the other person reacts to the perceived stimulus? Normally, we are using our hands or eyes to point at different objects in our environment. Now, the other person could either point or gaze at the same object. It is still unclear whether hand or eye movements are more suitable to perform this type of tasks. In addition, it is an open question whether it does matter how one reacts to a perceived movement, either by a matching or mismatching response action? This paper focusses on a possible coupling effect of response type and perceived stimulus, either by matching mismatching the response type.

Gaze-following plays a crucial role in social interaction both in humans and animals [1,2] Gaze-following can be already observed from very early on in the development of children, for example in joint attention as the capacity to modify other persons attention on the basis of gaze behavior and in the development of mindreading as the capacity to adequately ascribe mental states to others in order to explain or predict their behavior [3]. Based on a simple gaze-shift, humans can deduce the motivation, desires and preferences of their interaction partners and redirect their attention accordingly [4–7]. From an evolutionary perspective, the morphology of the human eye is particularly interesting as the high contrast between the white sclera and dark iris compared to other non-human primates could have facilitated to send messages over long distances as a remote communication system [8]. Teleologically, this unique morphology might be the foundation of the “social gaze”, a key issue for social interaction [9].

Besides the eyes, also our hands contribute to sending and reacting to signals and therefore redirecting attention. Interestingly, children respond stronger to pointing hands compared to gaze-shifts [10–12]. By pointing to specific objects, already 12-month-old infants share attention and emotions with others [13]. Seeing a pointing hand shifts the attention to the cued side, even if the stimulus is irrelevant to the task [14]. Observing hand actions triggers the simulation of the same actions [15] and facilitates congruent manual responses [16,17].

An important question refers to whether gaze cues have a higher influence on attentional redirection compared to non-biological cues, e.g., arrows [18–25], because it is unclear whether attentional shifts are differentially modulated by social or non-biological signals [17]. By using a modified Posner-task in a stimulus-response compatibility (SRC) task and investigating the effects of gaze-shifts on action control, we could show that participants who reacted to biological stimuli (i.e., moving eyes) showed faster and more accurate responses compared to conditions in which an object stimulus (i.e., a square in front of a face-like background) was presented and that incongruency costs were lower when interacting with a social compared to an object stimulus [26,27].

It is still under debate though, whether the redirection of attention is triggered by the social relevance of biological stimuli in general or whether there is a specific coupling or a compatibility effect between the type of the presented signal and the response type (e.g., responding with the eyes to a gaze-shift versus to a pointing hand). Elaborating this issue Crostella et al. [28] employed a simple joint attention task with different distractors. Participants had to respond to a color-changing fixation point with a leftward or a rightward movement (either saccades or hand movements). Distractors were a human face performing gaze-shifts, a hand or an arrow, which were pointing either to the same side as the side indicated by the color code or to the incongruent side. The results showed that the distraction by incongruent gaze-shifts impaired saccadic performance significantly more than by incongruent hand or arrow stimuli; in line with this, a distracting hand impaired pointing performance significantly more than distracting gaze or arrows.

However, in our understanding, this setting does not adequately probe interactions in a sufficiently naturalistic way as it is necessary to make use of biologically relevant deictic gestures as cues—not as distractors—in order to study real-life interactions as close to reality as possible. In addition, we wanted to examine whether there is a “coupling effect” between the presented stimulus and the response type. To investigate these issues, we modified our previous experimental setup [27] in two separate experiments. In the first experiment we investigated the influence of social (“gaze”) and non-social (“object”) stimuli on two different response types, e.g., performed gaze-shifts or hand-movements. We hypothesized that i) social stimuli would lead to faster responses independent of the response modality and independent of the given task compared to the object stimulus, and that ii) the eyes as response type respond fastest when reacting to the social stimulus (“gaze”) due to the postulated “coupling effect”. In the second experiment, we changed the stimuli by replacing the object stimulus with another social stimulus (“hands”) in order to systematically and consequently vary cue type and response type. Here, we assume to find coupling effects for both response types when responding to the physiologically matching stimulus (“gaze”,”hand”).

## Material and methods

In the following, we employ a specific terminology of “(stimulus type)-to-(response type)” to describe the different combinations of cueing stimuli and response types. Stimuli could either be presented by gaze-shifts (“gaze”), by objects (“object”) or by finger movements indicating button presses (“hand”); response types were either gaze-shifts (“gaze”) or button presses (“hand”); resulting in six different possible combinations, namely gaze-to-saccade, object-to-saccade, hand-to-saccade, gaze-to-button press, object-to-button press, hand-to-button press.

## Experiments

### General experimental setup

Both studies were performed in a quiet and dark room. Subjects sat on a chair in front of a LCD monitor at a distance of approximately 70 cm (viewing angle 5.7° x 3.7° for the presented stimulus). This distance was maintained constant between subjects by using an adjustable chin-rest. Participants were requested to position and keep their hands on two LumiTouch devices (Photon Control Inc., Burnaby, BC, Canada) with their index finger on the corresponding response button. For the manual responses, the time of pressing the button on the LumiTouch device was recorded. Eye movements were recorded by the eye-tracker system EyeLink1000 (SR Research Ltd. Kanata, ON, Canada). The movements of the right eye were recorded with a sampling rate of 1 kHz. The experiment was performed using Presentation® software (Version 0.70, http://www.neurobs.com). Procedures were approved by the local ethics committee of the Medical Faculty of University of Cologne. The stimuli were created with Poser 6 (e frontier America, Inc.), a software program for designing 3D animations or were borrowed from a previous experiment [27].

### Experiment 1

#### Experiment 1: Material and methods

32 healthy volunteers (16 males; mean age 25.31 years, standard deviation (SD) 4.75), all right-handed according to a standard handedness inventory [29], participated in this experiment. All volunteers had a normal or corrected to normal vision and were naïve to the purpose of the study. Prior to the experiment they provided their written informed consent to participate in the study.

**Fig 1:**
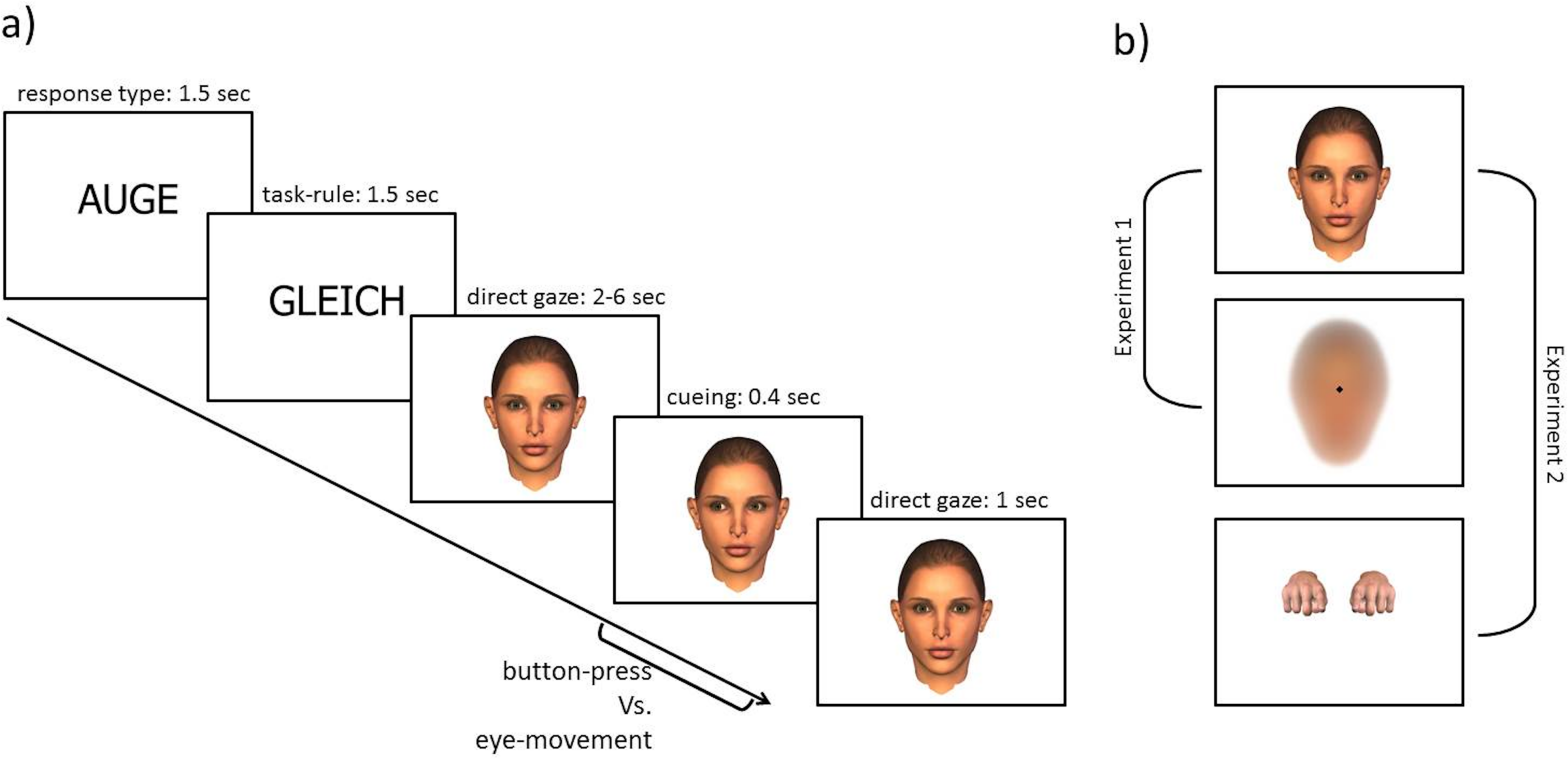
Experimental design. a) Exemplary depiction of event structure. After depicting the response type for the following block, the rule for the following trials is presented. b) Stimuli used in the study. In Experiment 1, the gaze and object stimulus was presented whereas in Experiment 2, the gaze and hand stimulus was used.

The experiment consisted of two parts for testing the two different stimuli. Both parts were presented in an alternating fashion from subject to subject. Each part consisted of 16 blocks with 12 trials each, leading to 384 trials for the whole experiment. In the beginning of every part, one of two response types to be used was indicated for 2.5 seconds by the words “HAND” (“hand”) or “AUGE” (“eye”) on the screen, respectively. Followed by the task, either “GLEICH” (“same”), representing the congruent (CON) rule in which the subject had to respond to the same side as the given directional cue (i.e., responding with their left index finger or looking to the left edge of the monitor frame to a stimulus pointing to the left) or “GEGEN” (“opposite”), representing the incongruent (INC) rule was shown. Subsequently, a block of 12 trials started either with the social or the non-social stimulus [27] For the social stimulus, a virtual character performing gaze shifts was used, whereas in the non-social stimulus, a small square of the same size as a pupil of an eye of the displayed agent was presented and displaced toward the left or right from a central starting position in front of a face-like background. The stimuli were matched for equivalent offsets in pixel coordinates as well as equivalent timing in the different conditions (see Fig 1A).

The stimuli were animated by applying the following procedure with the presentation of three different images: the first image (lasting 2 to 6 seconds, uniformly jittered) was replaced by a second image, lasting 10 ms, depicting a shift of the eyes (“gaze”) or diamond-like shape (“object”) for 3 pixel (relative to the starting position) to the left or right side, respectively. The third image presented the final position of the eyes or diamond, lasting for 400 ms (6px relative to the starting position). This procedure led to a convincing presentation of a gaze shift. At the end of 12 trials, a new block was presented, again initiated by the requested task (“same” versus “opposite”). In the second part of the experiment, the response type (“gaze” versus “hand”) switched. The order of the two parts was counterbalanced across subjects.

This experimental structure led to a 3-factorial 2 x 2 x 2 within-subject design with the factors STIMULUS (gaze versus object), TASK (congruent (CON) versus incongruent (INC)), and RESPONSE (gaze versus hand)(see Fig 1B).

#### Experiment 1: Data analysis

For the data analysis, only reaction times of correct trials were considered, and only trials with response latencies between 100ms and 1000ms were included in the analysis. The percentage of excluded trials was smaller than 1% of the total trials and thus was not further analyzed. It seems that the object stimulus led to slower RTs in the saccadic condition, compared to the gaze stimulus. However, this small amount of excluded trials (<1%) does not give any information about the performance and thus were not further analyzed. In addition, saccadic responses had to be on an end position at least 200px apart from the start position or more to ensure a gaze shift. All other trials were excluded from the data analysis. To avoid any task-switch and post-error slowing effects, the first trial of every block and one subsequent trial after an error trial, were excluded from the analysis (error-rate 2.82%). Processing of raw data of eye-tracking and subsequent data analysis were conducted in Python (Version 2.7.5), the R language of statistical computing (R Development Core Team (2011), Version 3.1.0, http://www.R-project.org) and SPSS (IBM Corp. Released 2012. IBM SPSS Statistics for Windows, Version 21.0. Armonk, NY: IBM Corp.).

#### Experiment 1: Results and discussion

**Fig 2:**
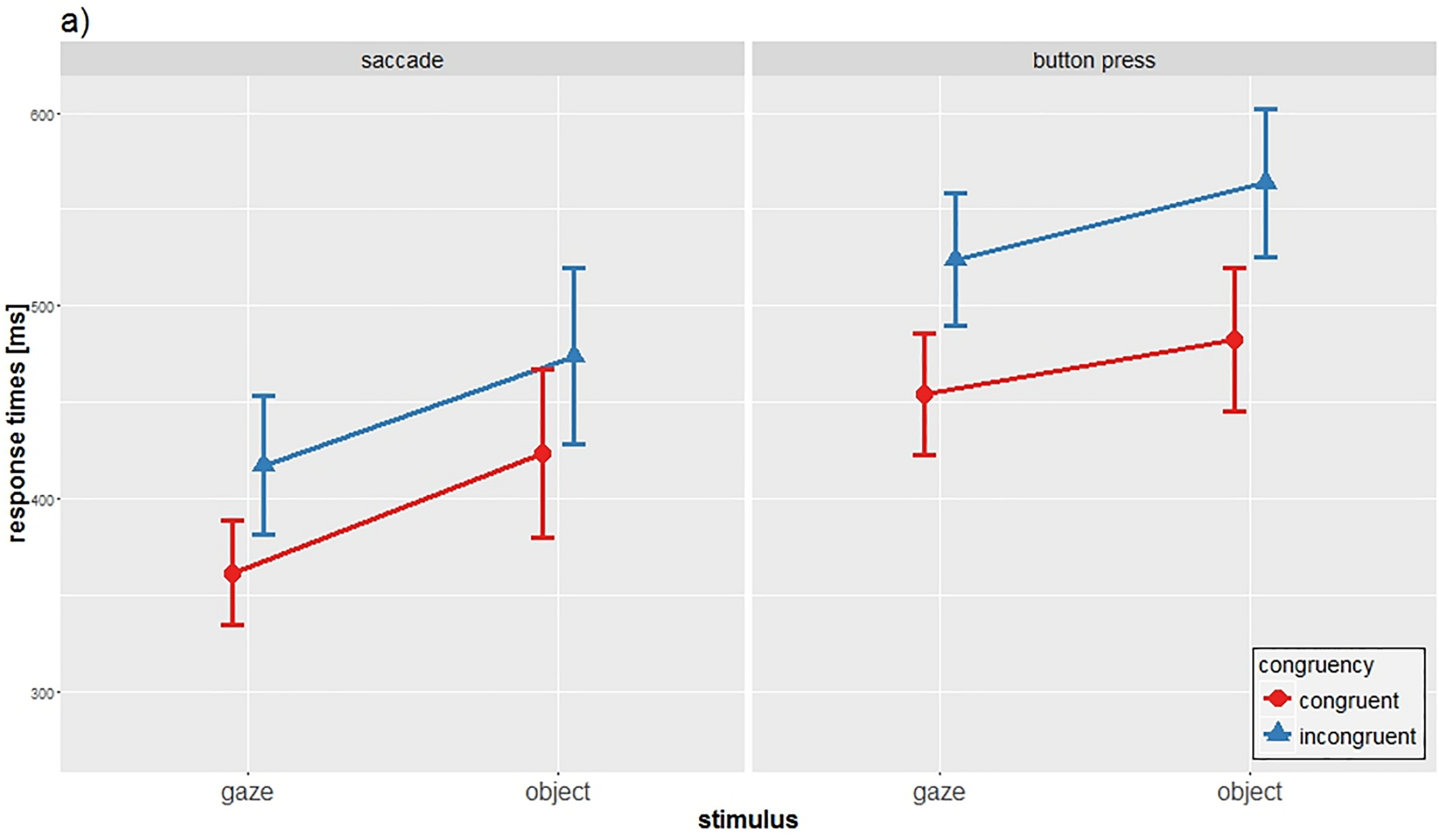
Main results of experiment 1. a) Mean reaction times and SD of the two response types for each combination of stimulus and condition.

Reaction times were aggregated by their median scores and analyzed using a three-way repeated measures ANOVA (see Fig 2).

The analysis revealed significant main effects for STIMULUS (*F*(1,31) = 50.57, *p*<0.001, η^2^ = 0.62), TASK (*F*(1,31) = 83.14, *p*<0.001, η^2^ = 0.728) and RESPONSE (*F*(1,31) = 76.86, *p*<0.001, η^2^ = 0.713). Responding to the gaze stimulus led to faster responses compared to the object stimulus, independent of TASK and RESPONSE (gaze: mean = 439,36ms; object: mean = 486.17ms). Also, CON reactions lead to faster responses compared to INC reactions (CON: mean = 430.68ms; INC: mean = 494.84ms). Eyes as response type show faster reactions than hands independent of TASK and STIMULUS (saccade: mean = 419.34ms; button press: mean = 506.19ms). Significant interactions were found for STIMULUS x RESPONSE (*F*(1,31) = 4.43, *p* = 0.043, η^2^ = 0.125) and for TASK x RESPONSE (*F*(1,31) = 4.46, *p* = 0.043, η^2^ = 0.126). The interaction of TASK x STIMULUS was not significant (*F*(1,31) = 0.15, *p* = 0.705, η^2^ = 0.005). Also the three-way interaction of STIMULUS x TASK x RESPONSE was not significant (*F*(1,31) = 1.53, *p* = 0.225, η^2^ = 0.047).

The significant interaction STIMULUS x RESPONSE suggested a possible “coupling effect” for matching stimulus and response types and was further assessed by conducting post-hoc *t*-tests. Gaze-to-saccade showed significant faster reactions than the object-to-saccade condition (mean gaze-to-saccade: 389.67ms, mean object-to-saccade: 449.00ms, difference: 59ms) (*t*(31) = 5.55, *p*<0.001). Hands also reacted significantly faster to gaze-stimuli than to object-stimuli (mean gaze-to-button press: 489.04ms, mean object-to-button press: 523.34ms, difference: 34ms) (*t*(31) = 5.24, *p*<0.001). The RT differences within a response modality show that saccadic responses seem to differentiate more between the two types of stimuli in comparison to manual responses.

Results from the first experiment reproduce the findings of the experiment conducted by Schilbach et al. [27]: They suggest that the social stimulus leads to faster responses in both response types. In addition, they demonstrate that responding with a matching response type to the presented stimulus (i.e., gaze-to-saccade) induces a “coupling effect” that leads to significant faster reaction times compared to all other conditions (object-to-saccade, gaze-to-button press, object-to-button press), meaning that gaze-to-saccade seems to be a special condition within this experiment. It has to be mentioned that in this experiment, only the eyes as response type could react to a matching stimulus and thus benefit from a possible “coupling effect”. The hands however did not have the opportunity to react to an adequate stimulus. In the second experiment, we exchange the object stimulus with a hand stimulus to ensure that both response types could react to a matching stimulus to benefit from the “coupling effect”.

### Experiment 2

#### Experiment 2: Material and methods

32 right handed, healthy volunteers (17 males, mean age 24.94 years, SD 3.61), participated in this experiment. Again, all participants were naïve with regard to the purpose of the experiment and had not participated in experiment 1.

To reduce the duration of the experiment, we substantially shortened experiment 2 to 192 trials instead of 384 trials from experiment 1. Setup, procedure and data analysis were identical to experiment 1. The percentage of excluded trials was smaller than 1% of the total trials and thus was not further analyzed. Again, no trials were excluded when responding with button-presses. We compared the effect size for the main effect RESPONSE and TASK and the interaction RESPONSE x TASK to make sure that both experiments were comparable. Setup, procedure and data analysis were identical to experiment 1.

The specific purpose of this experiment was to clarify the questions, first, whether a combination of matching stimulus and response type in general leads to faster responses, and second, whether gaze-to-saccade leads to faster responses compared to hand-to-button press.

To answer this question, a stimulus depicting a pair of hands was presented instead of the object stimulus (see Fig 1B). The designed hands perform finger movements corresponding to button presses, i.e., the index finger was pushing an imaginary button. With this setup, the participants could react with both response types to a matching stimulus, showing the same action as the movement of the participant. This design results in a 3-factorial design with the within-subject factors STIMULUS (gaze-shift versus hand movement), TASK (CON versus INC), and RESPONSE (saccade versus button press).

We hypothesized that responding to a stimulus with the matching response type (gaze-to-saccade, hand-to-button press) would lead to faster responses compared to a mismatch combination of stimulus and response type (hand-to-saccade, gaze-to-button press). In addition, we assumed that gaze-to-saccade leads to the fastest responses compared to all other combinations, in particular compared to hand-to-button press due to the higher importance in the development of social interaction.

#### Experiment 2: Results and discussion

The error-rate for experiment 2 was 0.798%. A three-way repeated-measures ANOVA was conducted. The analysis revealed significant main effects for TASK (*F*(1,31) = 124.26, *p*<0.001, η^2^ = 0.8) and RESPONSE (*F*(1,31) = 39.06, *p*<0.001, η^2^ = 0.558), but not for STIMULUS (*F*(1,31) = 0.03, *p* = 0.856, η^2^ = 0.001).

**Fig 3:**
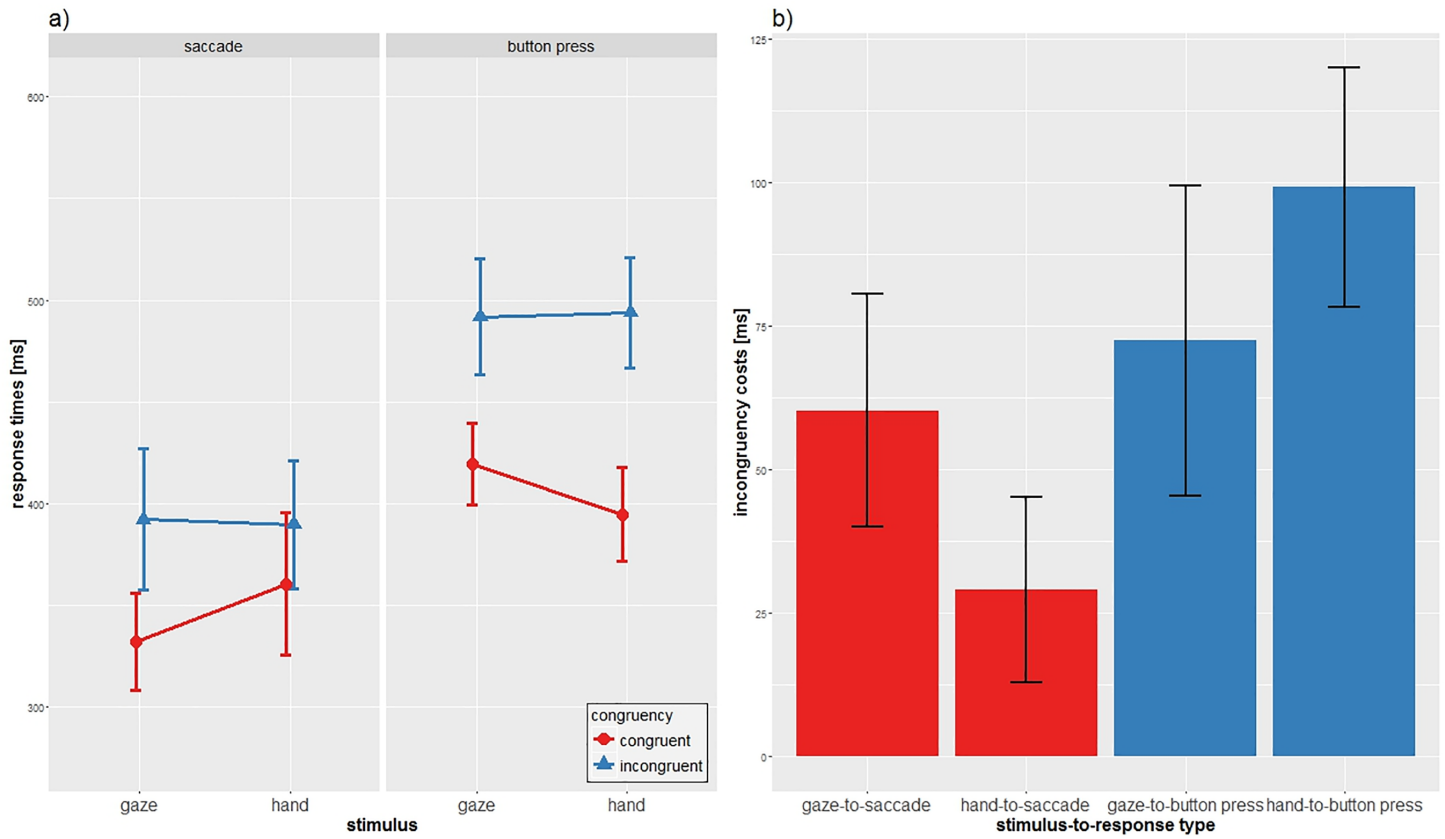
Main results of experiment 2. a) Mean reaction times and SD of the two response types for each combination of stimulus and condition. b) Incongruency costs and SD of the different combination of stimulus and response type.

CON conditions always led to faster responses compared to INC conditions (CON: mean = 376.66ms, INC: mean = 441.87ms) independent of STIMULUS and RESPONSE. Eyes as RESPONSE reacted faster compared to hands on average (saccade: mean = 368.60ms; button press: mean = 449.93ms). Both STIMULI, gaze and hand, led to similar response times independent of the task and response type (gaze: mean = 408.85ms, hand: mean = 409.68ms) which is represented in the non-significance of the main effect stimulus (see Fig 3A).

Significant two-way interactions were found for STIMULUS x RESPONSE (*F*(1,31) = 4.75, *p* = 0.037, η^2^ = 0.133) and for RESPONSE x TASK (*F*(1,31) = 16.60, *p*<0.001, η^2^ = 0.349), whereas the interaction for STIMULUS x TASK was not significant (*F*(1,31) = 0.04, *p* = 0.842, η^2^ = 0.001). Importantly, the analysis also revealed a three-way interaction between STIMULUS, RESPONSE and TASK (*F*(1,31) = 10.61, *p* = 0.003, η^2^ = 0.255).

Due to the long duration of experiment 1, we decided to shorten experiment 2 to prevent any fatigue effects. To test if this shortage of trials alters the previously found effects, we performed a between-experiment ANOVA with TASK as within-subject factor for both response types separately. For the saccadic responses, ANOVA yielded no significant differences for the interaction EXPERIMENT x TASK (*F*(1,62) = 0.774, *p* = 0.382, η^2^ = 0.012). This was also true for the RESPONSE “button press” (TASK x EXPERIMENT: *F*(1,62) = 0.774, *p* = 0.382, η^2^ = 0.012). In addition, we compared the effect sizes of the main effects for RESPONSE and TASK in both experiment (Exp.1: RESPONSE: η^2^ = 0.713, TASK: η^2^ = 0.728; Exp. 2: RESPONSE: η^2^ = 0.558, TASK: η^2^ = 0.8). In summary, our results suggest that experiment length or different stimuli do not influence the statistical power of our results regarding the factors RASK and RESPONSE.

To further investigate the three-way interaction, we split the data and conducted two-way ANOVAs.

First, to compare the natural gaze-following behavior (gaze-to-saccade) with the contrasting hand-to-button press condition, we conducted a two-way ANOVA with STIMULUS and RESPONSE for the CON task only. We found a significant main effect for RESPONSE (*F*(1,31) = 23.124, *p*<0.001), which was due to significantly faster responses of saccades compared to hand (*t*(31) = 4.81, *p*<0.001).

The interaction STIMULUS x RESPONSE was significant (*F*(1,31) = 12.142, p = 0.001) as indicated by diverging RT differences for the two stimuli within response modalities. The gaze stimulus had a greater impact on both response types compared to the hand stimulus (mean gaze-to-saccade: 332.02ms, mean gaze-to-button press: 419.36ms, difference: 87ms, mean hand-to-saccade: 360.52ms, mean hand-to-button press: 394.73ms, difference: 34ms). Results of the RT differences show, in line with experiment 1, that eyes as response modality seem to differentiate more between the two stimuli in comparison to manual responses.

When comparing gaze-to-saccade with hand-to-saccade, gaze-to-button press and hand-to-button press, post-hoc *t*-tests revealed that gaze-to-saccade was significant faster compared to all other conditions (gaze-to-saccade, gaze-to-button press: t(31) = 6.66, p<0.001; gaze-to-saccade, hand-to-saccade: t(31) = 3.23, p = 0.003; gaze-to-saccade, hand-to-button press: t(31) = 5.66, p<0.001). All other comparisons also yielded significant differences (hand-to-saccade, hand-to-button press: t(31) = 2.11, p = 0.043; hand-to-saccade, gaze-to-button press: t(31) = 3.50, p = 0.001; gaze-to-button press, hand-to-button press: t(31) = 2.20, p = 0.035).

Second, we conducted a two-way ANOVA with STIMULUS and RESPONSE for the INC task. We found a significant main effect for RESPONSE (*F*(1,31) = 45.16, *p*<0.001) due to faster responses of gaze compared to hand (*t*(31) = 6.72, *p*<0.001). However, we did not find a significant interaction of STIMULUS x RESPONSE (*F*(1,31) = 0.132, *p* = 0.718), since both responses did not differentiate between the two stimuli (gaze-to-saccade versus hand-to-saccade: *t*(31) = 0.292, *p* = 0.773; gaze-to-button press versus hand-to-button press: *t*(31) = 0.192, *p* = 0.849). It could be possible that suppressing the inner motivation to follow the perceived movement overlays possible “coupling effects”. The three-way interaction can thus be explained by the fast responses of the gaze-to-saccade condition in the CON task.

For the analysis of the incongruency costs, i.e., the reaction time differences between the congruent and incongruent condition within a response type, *t*-test were conducted. We performed paired samples *t*-tests for RESPONSE, showing significantly smaller incongruency costs for the saccade condition (mean: 44.66ms) compared to the button press condition (mean: 85.77ms) (*t*(31) = 4.074, p<0.001). The paired samples *t*-test for STIMULUS showed no significant difference (*t*(31) = 0.2, p = 0.842).

Results demonstrated that the gaze-to-saccade condition had significantly higher incongruency costs than the hand-to-saccade condition (mean gaze-to-saccade: 60.25ms, hand-to-saccade: 29.08ms, *t*(31) = 2.44, *p* = 0.020). The comparison of gaze-to-button press and hand-to-button press was not significant (mean gaze-to-button press: 72.41ms, hand-to-button press: 99.13ms, *t*(31) = 1.72, *p* = 0.096). The comparison of the social stimuli with both response types (gaze-to-saccade vs gaze-to-button press) shows no significant difference (*t*(31) = 0.78, *p* = 0.443). However, the hand-stimulus elicit lower incongruency costs when the response type is eyes compared to response type hands (hand-to-saccade versus hand-to-button press, *t*(31) = 6.50, *p*<0.001). When the eyes or hands interact with their matching stimuli, the gaze-to-saccade condition has significantly less incongruency costs than the hand-to-button press condition (*t*(31) = 2.77, *p* = 0.009). In the mismatching condition, i.e., the response type does not match the stimuli, the hand-to-saccade condition shows significantly less incongruency costs than the gaze-to-button press condition (*t(31) =* 2.72, *p* = 0.011).

In summary, similar to results from experiment 1, gaze-following responses were the fastest responses independent of task and stimulus. In addition, gaze-to-saccade condition led to the fastest responses compared to all other combinations (hand-to-saccade, gaze-to-button press, hand-to-button press).

Importantly, none of the conducted ANOVA had a significant main effect for stimulus, what might indicate that gaze and hand stimuli were perceived equivalently salient. However, while incongruency costs were generally lower for saccadic responses than for manual responses, incongruency costs were particularly low for responses in the hand-to-saccade-condition and were highest for the hand-to-button press condition. It is also worth mentioning that the combination of response type and matching stimulus (hand-to-button press, gaze-to-saccade) had higher incongruency costs compared to the corresponding mismatch-combination type (i.e., gaze-to-button press, hand-to-saccade).

This effect could be explained by mimicry or a coupling effect. When the stimulus matches the response type, the intrinsic motivation to mimic the perceived action leads to faster responses in the congruent condition compared to the mismatching combinations, where this advantage of mimicry is reduced. In the incongruent condition, matching stimulus and response do not benefit from such a coupling effect because the reflexive congruent response has to be stopped and an incongruent motor response has to be initialized. In contrast, a mismatch combination of stimulus and response type in the incongruent condition does not require a comparably high additional effort. Thus the incongruency costs are smaller for the mismatching conditions than for the matching combinations.

## General discussion

The main goal of this study was to demonstrate the unique feature of gaze-following behavior. In two experiments, we investigated the interaction of two response types with different sets of stimuli. The first experiment investigated the influence of social and non-social (gaze versus object) stimuli on the reaction times of two different response types (gaze versus hand). Results show that interacting with social stimuli led to faster responses for both response types. This finding is in line with previous studies [26,27]. In addition, results show that the gaze-to-saccade combination, thus when the stimulus matches the response type, benefits from a “coupling effect”. The second experiment enhances the first experiment by adding a matching stimulus for the response type hand in such a way that both response types have the opportunity to interact with a matching stimulus. Both response types show faster responses when interaction with visually comparable stimulus. However, this could only be found in the congruent condition.

The results of the two experiments provide evidence for distinct, response type dependent cognitive control mechanisms for gaze-based versus hand-based actions in social interaction.

First, the results of Experiment 1 showed that eyes and hands as response types differentiate between a social and non-social stimulus. These findings are in line with previous findings. It seems that the participants’ performance benefits from the presence of the social stimulus, maybe due to the idea of social facilitation [30], which suggests that the mere presence of another person, or, in our case, the interaction with a virtual other, increases physiological arousal that could lead to better performances. It could also be possible that the face might be perceived as more behaviorally relevant and could thereby improve response processes, e.g. modulate inhibitory processes [31]. It is worth mentioning that the eyes respond to both stimuli significantly faster compared to the hands. In addition, the eyes seem to be less influenced by the tasks compared to the hands.

It is not surprising that the gaze-to-saccade condition led to the fastest responses. Gaze-following is a well-known and well described phenomenon that can be found already in newborn babies. In contrast, an object stimulus does not seem to trigger a comparatively reflexive and rapid shift of attention towards the given direction. An object-stimulus does not describe a sufficiently naturalistic situation and thus the motivation to follow this directional cue is diminished [18–25,32].

As already mentioned, our results showed that eyes generally reacted faster than hands. This could be explained by the idea of “different neural domains”, of perceiving of and reaction to different stimuli [33]. For the eyes, the two properties of perceiving and sending signals are represented in the same “eye-network”. However, the hands do not integrate these two properties in their “hand-network”. Perceiving external information is dependent on the “eye-network” and just sending information lies in the “hand-network”. Therefore, reacting with hands to visual stimuli would require the recruitment of two distinct networks, whereas the eyes just need to activate one network. This could lead to general slower responses for the hands.

Both response types share several similarities. Both prefer responding to naturalistic stimuli (e.g., saccade and button presses) compared to the object stimulus of the first experiment. To react to these naturalistic or human stimuli might be well-known and thus fast processed. In comparison, responding to an object might be an unfamiliar behavior which therefore leads to slower responses. In addition, both response types prefer responding to a matching compared to a mismatching stimulus in the congruent condition. This finding is in line with studies about mimicry and imitation [16,34], showing shorter RTs when mimicking a matching action in comparison to longer RTs when performing an inverse, or mismatching action.

In both experiments, the incongruent task has a significant interference effect on movement execution. Interestingly, observing an incongruent movement led to a similar response pattern in both response types. In the incongruent condition, both response types do differentiate between the social and non-social stimuli (saccade and object) but not between the two social stimuli (saccade and button press). This finding could illustrate that in the incongruent condition, both response types differentiate the stimuli of their social valence and not of their depicted bodypart or movement and thus, there is no effect of stimulus in the incongruent condition in the second experiment. Both stimuli seem to have the same social valence. In addition, it seems that the additional workload of the incongruent task also overlays the matching or mismatching benefit or disadvantage of the stimulus to the response type.

The results of both experiments show that the influence of social stimuli may change under specific circumstances, such as, for example, when different response types are used to make a response. In addition, the results may suggest that the construction of joint attention, one of the milestones of social interaction [35], is much more dynamic than expected, which is in concordance with previous research [20,31,36,37]. However, our study extends previous findings by showing that not only bodyparts as distractor but also as effector stimulus can modulate attentional shifts [28]. In this study, we have a possible limitation concerning the used stimuli in experiment 2 (gaze and hand movements) in regard of redirecting attention. We assume that the strength of redirecting for the stimuli was comparable in the experiment. However, this study was not focusing on spatial attention but on the similarity of perceived movements and response actions, therefore this possible confound should not alter the reported findings.

For future prospects, we could explore the network of the integration of bodypart specific mapping. Indeed, areas involved in face, gaze, hand, and even full body perception may be involved in this kind of social attention task [38,39]. To investigate the influence of the probability of two different networks for eyes and hands, it would be possible to use tactile instead of visual stimuli. This could disentangle the question if the eyes are faster than the hands in general or if these faster reactions are dependent on the way the stimulus is presented.

## Conclusion

The main finding of this study is that different biological stimuli do have different strength in redirecting one’s attention. However, the behavioral results for the perceived stimulus are also dependent on the bodypart that reacts to the perceived stimulus. When the stimulus matches the response type, a “coupling effect” can be observed which leads to faster reaction times compared to a mismatch condition. In addition, saccadic responses, in comparison to button presses, seem to be less influenced by the task, meaning that incongruent trials as less distracted for eyes than hands.
